# The epidemiology of hereditary spastic paraplegia and associated common mental health outcomes in England and Northern Ireland

**DOI:** 10.1186/s13023-025-03849-3

**Published:** 2025-07-01

**Authors:** Harini Jeyakumar, Joht Singh Chandan, Krishnarajah Nirantharakumar, Siang Ing Lee

**Affiliations:** 1 Department of Applied Health Sciences, University of Birmingham, Birmingham, UK; 2 Department of Population Health Sciences, Kings College London, London, UK

**Keywords:** Hereditary spastic paraplegia, Epidemiology, Prevalence, Incidence, Mental health outcomes, Depression, Anxiety

## Abstract

**Background:**

Hereditary Spastic Paraplegia (HSP) is a rare genetic neurological disorder that causes progressive spasticity and weakness in the lower limbs. This study aims to describe the prevalence and incidence of HSP and examine common mental health outcomes (depression and anxiety) in HSP patients in England and Northern Ireland.

**Methods:**

This retrospective cohort study used CPRD Aurum primary care data from 1 January 2000 to 31 December 2021. Annual cross-sectional and cohort studies were conducted for yearly prevalence and incidence of HSP. Common mental health outcomes were examined with a 1:4 matched cohort (age+/−1 year, sex, general practice). Descriptive analysis and logistic regression assessed the characteristics of the HSP cohort and baseline depression and anxiety. Cox regression assessed the hazard of new diagnosis of depression and anxiety.

**Results:**

The overall cohort included 31,302,579 patients; the matched cohort included 1455 HSP patients and 5726 control non-HSP patients. Patients who were male (adjusted odds ratio [aOR] 1.45, 95% CI: 1.31–1.61), of White ethnicity (lower odds in all other ethnicity) and from most other geographical areas compared to London had higher odds of a HSP diagnosis, with the highest odds for North East (aOR 3.51, 95% CI: 2.73–4.50) and Northern Ireland (aOR 3.15, 95% CI: 1.62–6.16). HSP prevalence increased from 2.83 per 100,000 population (95% CI: 2.49–3.20) in 2000 to 6.27 per 100,000 population (95% CI: 5.83–6.73) in 2021. HSP incidence remained stable from 0.12 per 100,000 person-years (95% CI: 0.06–0.22) in 2000 to 0.29 per 100,000 person-years (95% CI: 0.20–0.40) in 2021. HSP patients had higher odds of baseline pre-existing depression (aOR: 1.74, 95% CI: 1.47–2.06) and anxiety (aOR: 1.31, 95% CI: 1.08–1.60); and higher hazard for new diagnosis of depression (adjusted hazard ratio [aHR]: 1.57, 95% CI: 1.26–1.96) and anxiety (aHR: 1.41, 95% CI: 1.12–1.76).

**Conclusion:**

This first descriptive epidemiological study for HSP in England and Northern Ireland, demonstrated the utility of primary care routine health records for studying rare diseases. The higher rates of common mental health conditions in HSP patients illustrated the importance of access to mental health support.

## Background

Hereditary Spastic Paraplegia (HSP) is a heterogenous group of monogenic neurological disorders primarily affecting the spinal cord, leading to progressive spasticity and weakness in the lower limbs, causing mobility issues [1]. The mode of inheritance includes autosomal dominant, autosomal recessive, X-linked and some cases are sporadic [1, 2]. A genetic diagnosis is not made in 51–71% of HSP patients, however more than 80 genetic types of HSP has been reported to date [1–3]. The Harding classification categorises HSP into pure (lower limb weakness and spasticity) and complex (additional neurological symptoms) [4].

Globally, the prevalence of all forms of HSP is estimated at 3.6 per 100,000 people, with regional prevalence variations that underscore genetic and methodological diversity across studies [5]. For instance, Norway reports a prevalence of 7.4 per 100,000, while Spain estimate lower prevalence rates of 2.24 per 100,000 respectively [6]. In Ireland, McMonagle et al. (2002) estimated a prevalence of 1.27 per 100,000 for pure autosomal dominant HSP [7]. As such, HSP is considered a rare disease, as defined in the UK’s Rare Disease Framework with a prevalence of less than 1 in 2000 [8]. To date, there is no study reporting the epidemiology of HSP in the UK.

Most epidemiological studies utilised national or regional patient registries, highlighting its importance in studying rare disease [9, 10]. There is currently no national HSP registry in the UK. The National Disease Registration Service (NDRS) in England intends to include more rare diseases, one option is to utilise routine health records such as hospital or primary care records. Although NDRS can access Hospital Episodes Statistics, there is currently no established data flow from primary care to NDRS [8]. As HSP is an outpatient-based condition, using hospital-based records as the data source may underestimate the prevalence. Therefore, using research ready primary care routine health records, such as the Clinical Practice Research Datalink to estimate the prevalence and incidence of HSP in the UK will provide a useful comparison when a national registry for HSP is established in the future [11].

In the UK, health care is free at the point of care [12]; 98% of the population is registered with a general practitioner, who is the gatekeeper for referrals to specialists in hospitals [11]. In the case of HSP, adult and paediatric patients presenting with related symptoms would be referred by their general practitioner to a neurologist or paediatrician to be assessed for a diagnosis. Following outpatient attendances, specialists send clinic letters to general practitioners, the diagnoses listed in the letters will usually be coded in the patients’ general practice records.

A recent UK survey showed over 90% of people living with rare diseases and their carers felt anxious, stressed or depressed [13]. HSP is characterised by progressive mobility impairments, and over time can result in a loss of independent walking ability, impacting quality of life [14, 15]. Balance and gait impairment can limit a person with HSP from participating in daily activity and social life and result in negative emotional and mental well-being [16]. Limited studies with small sample size has demonstrated an association of HSP with mental ill health, using self-assessment with validated questionnaires [15, 17]. For instance, Vahter et al. (2009) assessed depression prevalence in 48 HSP patients using the Beck Depression Inventory (BDI). Results showed 58% had depression, with 44% mild, 13% moderate, and 2% severe [18]. Depression scores correlated with reduced mobility, highlighting the impact of disease severity on mental health. A study conducted with patients from Brazil in 2014 reported that 36.6% of patients with SPG4-HSP had depressive symptoms (BDI score ≥ 11), in contrast to only 3.3% in the control group [19]. However, none were based in the UK, used recent data or used clinically diagnosed mental health conditions.

This research was initiated in response to a request from the UK HSP Support Group. Studying the epidemiology of HSP and it’s mental health impact is essential to help inform health service planning in the UK. This study aims to describe the prevalence and incidence of HSP and common mental health outcomes (depression and anxiety) in people living with HSP, using contemporary routine health records in England and Northern Ireland.

## Methods

### Study design

This is a retrospective cohort study from 1 January 2000 to 31 December 2021, using primary care routine health records in England and Northern Ireland. Annual cross-sectional and cohort studies were conducted between 1 January 2000 and 31 December 2021 to calculate yearly prevalence and incidence of HSP. The development of common mental health outcomes (depression and anxiety) was studied using a 1:4 matched cohort.

### Data Source

CPRD Aurum contains anonymised longitudinal primary care records from general practices using EMIS web® general practice patient management software in England and Northern Ireland, representing a broad cross-section of the population. As of December 2023, CPRD Aurum has data from 1771 general practices, encompassing over 46 million individuals, with 16 million current patients (24% of the population) [20]. The database captures information on patient demographics, diagnoses, and lifestyle factors [11].

### Inclusion/exclusion criteria

The study population included all patients registered with general practices contributing data to CPRD Aurum. To ensure data quality, general practices were eligible after 1 year of reaching data standards; patients were eligible if there were registered with the eligible general practices for at least one year. The first ever event of HSP ever recorded was used. Patients without a previous recorded diagnosis of HSP at the beginning of each 1-year study period were included in the yearly incidence studies.

For the common mental health outcome matched cohort study, HSP patients were matched to patients without a recorded diagnosis of HSP by age (+ / − 1 year), sex and general practice at a 1:4 ratio. Patients with pre-existing depression or anxiety were excluded from this analysis. The index date was the latest of general practice eligible date, patient eligible date, HSP diagnosis date or study start date (1 January 2000). Patients were followed up until the earliest of the following dates: common mental health outcome event, death, patient left practice, practice stopped contributing to the database and study end date (31 December 2021).

Data were extracted using the Data Extraction for Epidemiological Research (DExtER) 33 tool, from the December 2023 CPRD Aurum dataset [21].

### Variables

HSP, depression, and anxiety were ascertained by a recorded diagnosis in the patient’s general practice records, captured with SNOMED-CT codes (Appendix 1–3).

Age at index date was categorized into 0–16, 17–30 and thereafter as 10-year age bands. For the common mental health outcome study matched cohort, data was additionally extracted for body mass index (BMI) and smoking status; linked small area level data was obtained for patient level Index of Multiple Deprivation (IMD).

BMI was recategorized by World Health Organisation categories [22]. Smoking status were categorised as current, never/non-smoker, ex-smoker, and missing. Socioeconomic status was measured by patient level IMD deciles, with the first decile being the least deprived. [23]. Missing categorical data were represented as a separate category.

### Statistical analysis

Descriptive analysis characterized the overall cohort based on age at index date, sex, ethnicity, and geographical distribution; and additionally, BMI, smoking status and socioeconomic status for the HSP and matched-control cohort. Logistic regression examined the association of a HSP diagnosis with sex, ethnicity and geographical location in the overall cohort. As age at index date does not reflect age at diagnosis, it was not included in the logistic regression.

Prevalence and incidence are reported annually per 100 000 population and per 100 000 person-years, respectively, with 95% confidence intervals (CI) and presented in graphs. Annual HSP prevalence was calculated by dividing cumulative HSP cases by the total population, while incidence was determined by dividing new cases by person-years at risk.

Common mental health conditions are prevalent in the general population [24]. Therefore, for the matched cohort, we presented the prevalence of depression and anxiety at baseline and conducted a logistic regression examining the associations between HSP and baseline depression and anxiety. This was followed by a time-to-event analyses using Cox proportional hazards models, with time since cohort entry as the time scale, to estimate hazard ratios (HRs) for developing depression or anxiety after the index date. Both the logistic and regression analysis adjusted for age at index date, sex, ethnicity, BMI, smoking, and IMD.

### Patient and public involvement

This research was initiated in response to a request from the UK HSP Support Group. Members of the UK HSP Support Group was consulted on the research questions and outcomes to study. They emphasised the importance of understanding the epidemiology and mental health outcomes in HSP patients [25].

## Results

### Baseline characteristics

#### Overall study cohort

**Fig. 1:**
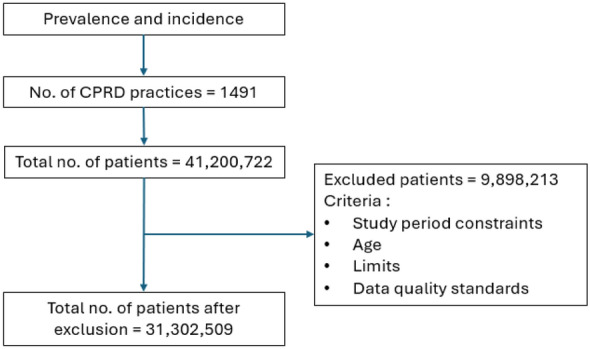
Flowchart of the patient selection for the overall cohort

A total of 31,302,579 patients were included in the prevalence and incidence study. (Fig. 1, Table 1). The largest age group in the overall study cohort comprised individuals aged 17–30 years (27.00%), with similar distribution across male (49.02%) and females (50.97). Majority of participants were of White ethnicity (50.09%), followed by Asian (7.22%), Black (3.72%), and Mixed or Multiple ethnic backgrounds (1.53%). Ethnicity data were missing for 36.10% of patients. Geographically, 99.68% of the cohort resided in England, with the highest distribution in London (23.93%), the South East (20.28%), and the North West (16.38%).

**Table 1:** Baseline characteristics of the overall cohort, the HSP patients and matched-control patients for the common mental health outcome study

Characteristics	Overall Cohort	HSP patients	Matched-control (non-HSP)
Total	31,302,579	1455	5726
Age at index date (in years), Mean (standard deviation)	32.41 (21.88)	42.89 (20.55)	42.23 (20.48)
Age at index date (in years), Median (interquartile range)	29.82 (18.22–45.46)	43.98 (27.71–58.99)	43.19 (27.33–58.29)
*Age categories at index date (in years), n (%)*			
0–16	7,294,737 (23.30)	179 (12.30)	721 (12.60)
17–30	8,452,976 (27.00)	231 (15.88)	944 (16.50)
31–40	5,650,446 (18.05)	216 (14.85)	881 (15.40)
41–50	3,394,679 (10.84)	258 (17.73)	1,004 (17.53)
51–60	2,541,562 (8.12)	225 (15.46)	858 (14.98)
61–70	1,796,853 (5.74)	206 (14.16)	822 (14.36)
71–80	1,276,892 (4.08)	110 (7.56)	397 (6.93)
81+	894,434 (2.86)	30 (2.06)	99 (1.73)
*Sex, n (%)*			
Male	15,345,362 (49.02)	843 (57.94)	3309 (57.80)
Female	15,956,367 (50.97)	612 (42.06)	2417 (42.21)
*Ethnicity, n (%)*			
Asian	2,260,175 (7.22)	84 (5.77)	295 (5.15)
Black	1,165,419 (3.72)	16 (1.10)	126 (2.20)
Mixed	480,303 (1.53)	8 (0.55)	50 (0.87)
Other	418,122 (1.34)	< 5	43 (0.75)
White	15,679,071 (50.09)	950 (65.29)	3407 (59.50)
Missing	11,299,489 (36.10)	393 (27.01)	1805 (31.52)
*Health authority of England and Northern Ireland, n (%)*			
East Midlands	859,392 (2.75)	27 (1.86)	104 (1.82)
East Of England	1,249,207 (3.99)	74 (5.09)	290 (5.06)
London	7,490,601 (23.93)	198 (13.61)	771 (13.46)
North East	830,694 (2.65)	92 (6.32)	364 (6.36)
North West	5,126,962 (16.38)	298 (20.48)	1174 (20.50)
Northern Ireland	101,704 (0.32)	8 (0.55)	32 (0.56)
South East	6,348,382 (20.28)	286 (19.66)	1,135 (19.82)
South West	3,610,276 (11.53)	197 (13.54)	773 (13.50)
West Midlands	4,555,971 (14.55)	235 (16.15)	923 (16.12)
Yorkshire & The Humber	1,099,650 (3.51)	40 (2.75)	160 (2.79)
Missing	29,740 (0.10)		
*Index of multiple deprivation, n (%)*			
1 (Least deprived)		123 (8.45)	567 (9.90)
2		123 (8.45)	543 (9.48)
3		123 (8.45)	525 (9.17)
4		131 (9.00)	571 (9.97)
5		125 (8.60)	488 (8.52)
6		125 (8.60)	575 (10.04)
7		149 (10.24)	564 (9.85)
8		154 (10.58)	553 (9.66)
9		170 (11.68)	576 (10.06)
10 (Most deprived)		191 (13.13)	634 (11.07)
11 (Missing)		41 (2.82)	130 (2.27)
*Body Mass Index (kg/m*^*2*^*), n(%)*			
Underweight (< 18.5)		57 (3.92)	133 (2.32)
Normal weight (18.5 – 24.9)		405 (27.84)	1465 (25.60)
Overweight (25–29.9)		311 (21.37)	1259 (22.00)
Obesity class I (30–34.9)		115 (7.90)	528 (9.22)
Obesity class II (35–39.9)		37 (2.54)	184 (3.21)
Obesity class III (> 40)		27 (1.86)	109 (1.90)
Missing		503 (34.57)	2,048 (35.77)
*Smoking Status*			
Current Smoker		264 (18.14)	1075 (18.77)
Never/Non-smoker		760 (52.23)	2827 (49.37)
Ex-Smoker		75 (5.15)	249 (4.35)
Missing		356 (24.47)	1,575 (27.51)

#### HSP cohort

**Fig. 2:**
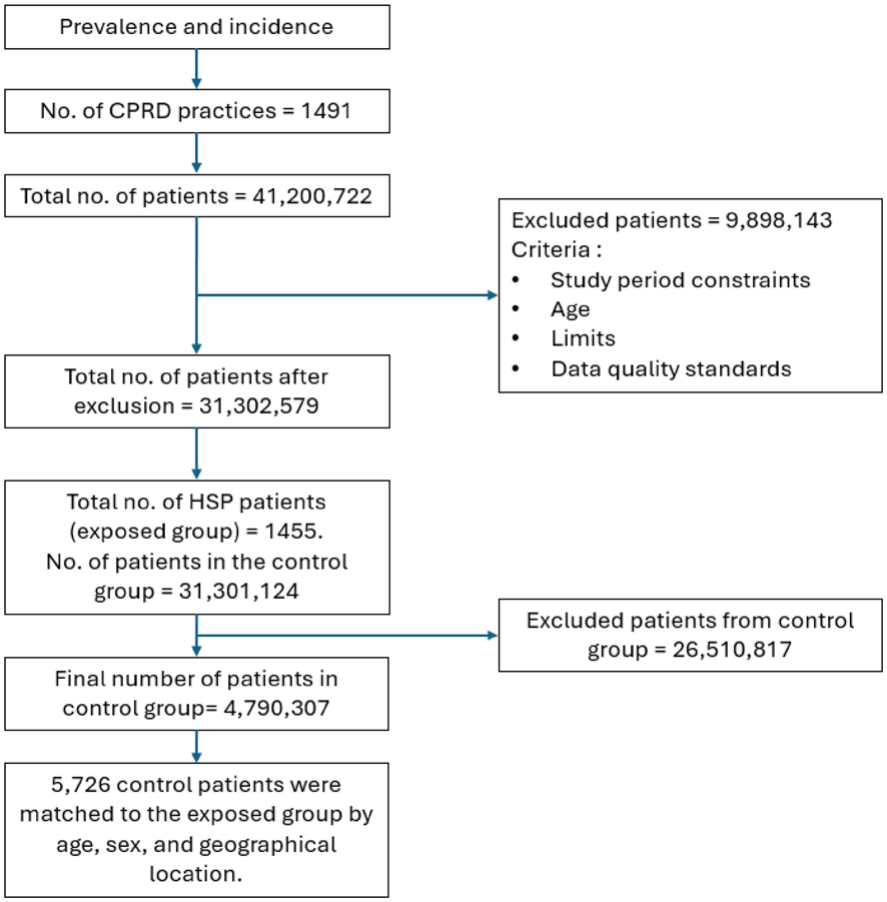
Flowchart of the patient selection for the mental health outcomes study

The common mental health outcome matched cohort study included 1455 HSP patients and 5726 matched-control patients (Fig. 2, Table 1). The mean age at index date for the HSP group was 42.89 years. There were higher proportion of male HSP patients (57.94%) and HSP patients of White ethnicity (65.29%). Within the CPRD Aurum HSP cohort, majority of HSP patients resided in the North West (20.48%), South East (19.66%), and West Midlands (16.15%). Regarding BMI, 27.84% of HSP patients were in the normal weight range and 34.57% had missing BMI data, 13.13% of the HSP cohort were in the most deprived decile, while smoking data revealed that 18.14% were current smokers and 52.23% were never smokers.

#### Logistic regression of HSP diagnosis and baseline characteristics in the overall cohort

Table 2 presents the logistic regression examining the association of a HSP diagnosis with sex, ethnicity and geographical area in the overall study cohort. Male patients had higher odds (adjusted odds ratio [aOR] 1.45, 95% CI: 1.31–1.61) of having a HSP diagnosis compared to female patients. Compared to patients of White ethnicity, patients of all other ethnicity had statistically significant lower odds of a HSP diagnosis. Compared to patients in London, patients from most other geographical areas had statistically significant higher odds of a HSP diagnosis, with the exception of East Midlands and Yorkshire and the Humber; the highest odds were observed in patients from the North East (aOR 3.51, 95% CI: 2.73–4.50) and Northern Ireland (aOR 3.15, 95% CI: 1.62–6.16).

**Table 2:** Logistic regression examining baseline characteristics associated with a diagnosis of HSP in the overall study cohort

	Unadjusted OR (95% CI)	Adjusted OR (95% CI)
*Sex*		
Female	Reference	Reference
Male	1.43 (1.29–1.59)	1.45 (1.31–1.61)
*Ethnicity*		
White	Reference	Reference
Asian	0.61 (0.49–0.76)	0.70 (0.56–0.88)
Black	0.23 (0.14–0.37)	0.30 (0.18–0.49)
Mixed or multiple ethnic background	0.27 (0.14–0.55)	0.32 (0.16–0.64)
Other	0.16 (0.06–0.42)	0.18 (0.07–0.49)
Missing	0.57 (0.51–0.64)	0.57 (0.51–0.64)
*Geographical areas*		
London	Reference	Reference
East Midlands	1.18 (0.79–1.77)	1.05 (0.70–1.57)
East of England	2.23 (1.71–2.91)	2.00 (1.53–2.61)
North East	4.17 (3.26–5.34)	3.51 (2.73–4.50)
North West	2.19 (1.83–2.62)	1.96 (1.64–2.35)
Northern Ireland	3.33 (1.71–6.50)	3.15 (1.62–6.16)
South East	1.71 (1.43–2.05)	1.57 (1.30–1.88)
South West	2.06 (1.70–2.51)	1.89 (1.55–2.30)
West Midlands	1.95 (1.61–2.35)	1.76 (1.46–2.13)
Yorkshire & The Humber	1.37 (0.97–1.92)	1.21 (0.86–1.70)

^*^Intersex and missing health authority all did not have HSP and were not included in the logistic regression. The adjusted logistic regression adjusted for sex, ethnicity and geographical areas

CI, confidence intervals

### Prevalence and incidence

**Fig. 3:**
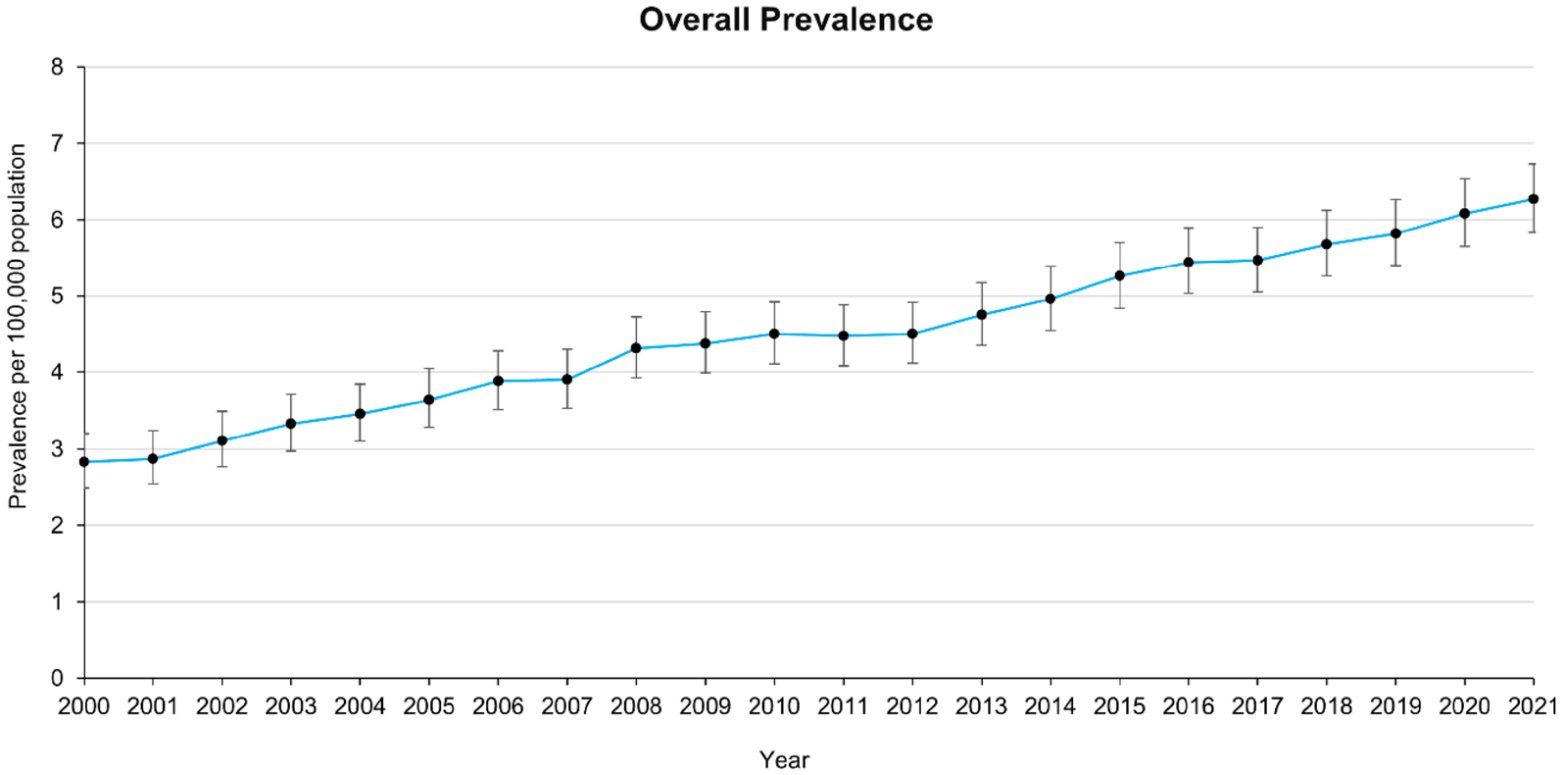
Trend showing HSP prevalence per 100,000 population

**Fig. 4:**
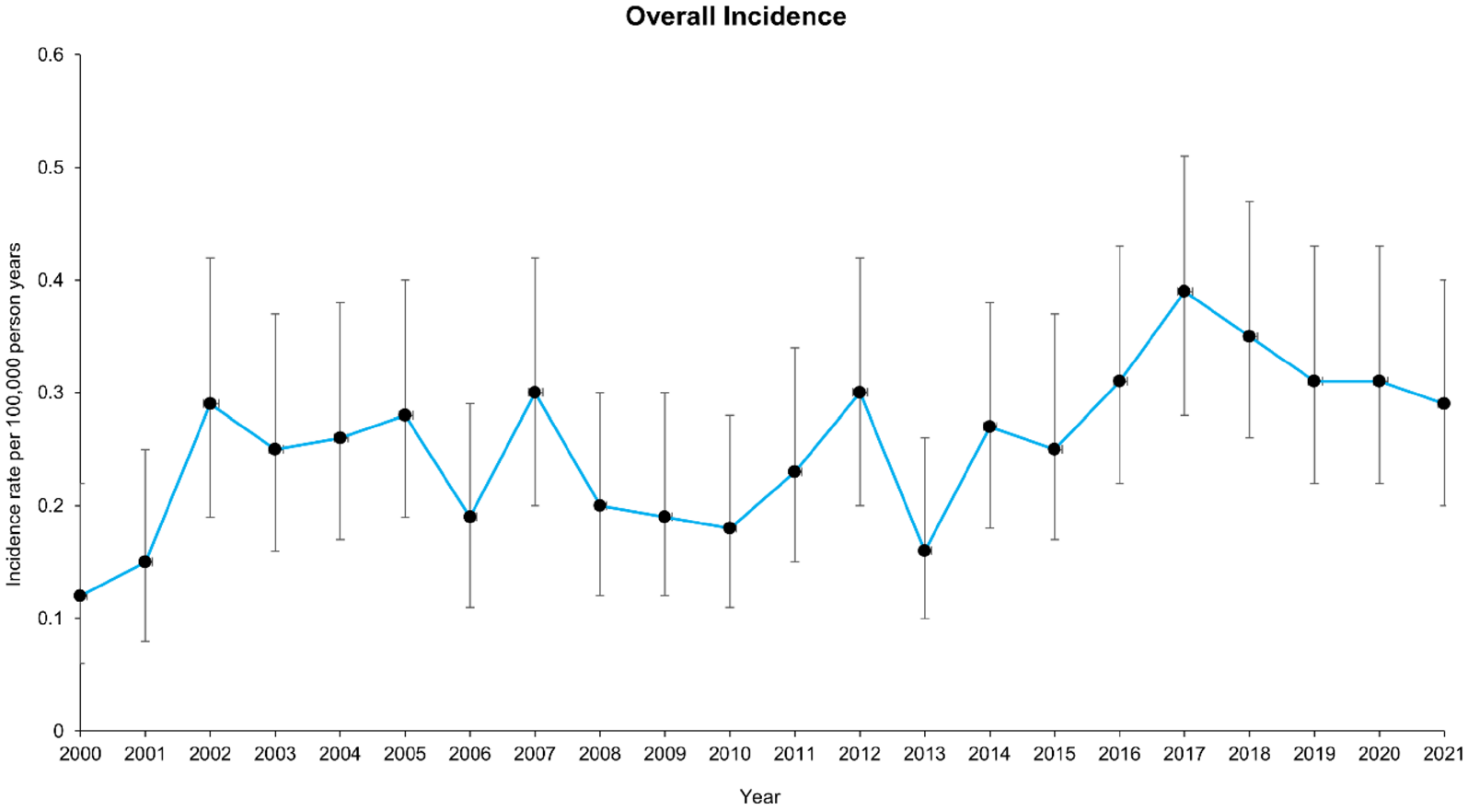
Trend showing HSP incidence rate per 100,000-person years

Prevalence showed a steady and statistically significant increase over the study period, rising from 2.83 per 100,000 population (95% CI: 2.49–3.20) in 2000 to 6.27 per 100,000 population (95% CI: 5.83–6.73) in 2021. The total number of diagnosed cases increased from 254 to 759 during this time, with narrowing confidence intervals over the years indicating increased precision in prevalence estimates. The prevalence and incidence trends are presented in Figs. 3 and 4 with full data tables available in the appendix 4 and 5. Conversely, the incidence of HSP remained relatively stable, ranging from 0.12 per 100,000 person-years (95% CI: 0.06–0.22) in 2000 to a peak of 0.39 per 100,000 person-years (95% CI: 0.28–0.51) in 2017 before declining slightly to 0.29 per 100,000 person-years (95% CI: 0.20–0.40) in 2021. Most year-to-year variations in incidence rates were not statistically significant, as evidenced by overlapping confidence intervals.

### Common mental health outcomes

#### Baseline pre-existing depression and anxiety

Prior to index date, the prevalence of pre-existing depression (19.18% versus 12.10%) and anxiety (11.89% versus 9.10%) was higher in patients with HSP compared to those without HSP (Table 3). Logistic regression also showed statistically significant higher odds of pre-existing depression (aOR: 1.74, 95% CI: 1.47–2.06) and anxiety (aOR: 1.31, 95% CI: 1.08–1.60) in patients with HSP compared to patients without HSP, after adjusting for age at index date, sex, ethnicity, socioeconomic status, BMI, and smoking status (Table 3, Appendix 6 for full model).

**Table 3:** Depression and anxiety at baseline and after diagnosis of HSP

	Depression		Anxiety	
	HSP	Matched-control	HSP	Matched-control
Total patients, n	1455	5726	1455	5726
*Baseline pre-existing common mental health conditions*				
Number of patients with outcome at baseline, n (%)	279 (19.18%)	693 (12.10%)	173 (11.89%)	521 (9.10%)
Adjusted OR (95% CI)	1.74 (1.47–2.06)		1.31 (1.08–1.60)	
*New diagnosis of common mental health conditions after HSP diagnosis*				
Total (excluding patients with outcome at baseline)	1176	5033	1282	5205
Number of outcomes	114 (9.69%)	318 (6.32%)	104 (8.11%)	316 (6.07%)
Person-years	8517	37,101	8974	37,862
Incidence rate (per 1000 person-years)	13.39	8.57	11.59	8.35
Adjusted HR (95% CI)	1.57 (1.26, 1.96)		1.41 (1.12, 1.76)	

CI, confidence intervals; HR, hazard ratios; HSP, hereditary spastic paraplegia; OR, odds ratio. Logistic and cox regression adjusted for age at index date, sex, ethnicity, socioeconomic status, BMI, and smoking status

#### New diagnosis of depression and anxiety

Excluding those with baseline depression and anxiety, a higher proportion of HSP patients had a new diagnosis of depression (9.69% vs 6.32%) and anxiety (8.11% vs 6.07%) after the index date, compared to patients without HSP. The incidence rate of depression was 13.39 versus 8.57 per 1000 person-years; and for anxiety was 11.59 vs 8.35 per 1000 person-years, in patients with and without HSP, respectively (Table 3).

In the fully adjusted Cox regression analysis (Table 4), HSP patients had a 57% higher hazard of developing depression (adjusted hazard ratio [aHR]HR: 1.57, 95% CI: 1.26–1.96) and a 41% higher hazard of developing anxiety (adjusted HR: 1.41, 95% CI: 1.12–1.76). Female patients had statistically higher hazard of depression (aHR: 1.70, 95% CI: 1.40–2.06) and anxiety (HR: 2.19, 95% CI: 1.80–2.67) compared to males. Compared to patients of White ethnicity, Asian patients were less likely to have a new diagnosis of depression (aHR: 0.43, 95% CI: 0.24–0.75) and anxiety (aHR: 0.60, 95% CI: 0.38–0.94). Compared to patients in the most affluent decile, patients in the most deprived decile had statistically higher hazard of a new diagnosis of depression (1.66, 1.08–2.54) and anxiety (2.10, 1.32–3.40).

**Table 4:** Cox regression for new diagnosis of depression and anxiety in patients with HSP compared to non HSP patients

	Adjusted hazard ratio (95% CI)	
	Depression	Anxiety
New diagnosis of depression	1.57 (1.26, 1.96)	–
New diagnosis of anxiety	–	1.41 (1.12, 1.76)
*Age at index date (in years)*		
0–16	Reference	
17–30	2.28 (1.53, 3.40)	1.74 (1.20, 2.53)
31–40	1.66 (1.09, 2.53)	1.34 (0.90, 2.00)
41–50	1.12 (0.73, 1.72)	0.88 (0.60, 1.31)
51–60	0.87 (0.54, 1.38)	0.61 (0.40, 0.96)
61–70	0.68 (0.42, 1.12)	0.50 (0.30, 0.80)
71–80	0.53 (0.24, 0.99)	0.34 (0.16, 0.70)
81+	0.90 (0.27, 2.98)	0.55 (0.13, 2.34)
*Sex*		
Male	Reference	
Female	1.70 (1.40, 2.06)	2.19 (1.80, 2.67)
*Ethnicity*		
White	Reference	
Asian	0.43 (0.24, 0.75)	0.60 (0.38, 0.94)
Black	0.54 (0.22, 1.32)	0.72 (0.34, 1.54)
Mixed	0.51 (0.12, 2.10)	0.55 (0.13, 2.26)
Other	0.84 (0.20, 3.44)	0.51 (0.07, 3.67)
Missing	0.77 (0.61, 0.98)	0.67 (0.52, 0.86)
*Index of multiple deprivation*		
1 (Least deprived)	Reference	
2	1.18 (0.74, 1.88)	1.52 (0.91, 2.53)
3	1.04 (0.63, 1.71)	1.44 (0.84, 2.50)
4	1.44 (0.91, 2.25)	1.77 (1.07, 2.92)
5	1.16 (0.72, 1.86)	1.33 (0.80, 2.28)
6	1.40 (0.88, 2.18)	1.40 (0.84, 2.32)
7	1.24 (0.78, 1.97)	1.64 (1.00, 2.68)
8	1.38 (0.90, 2.18)	1.46 (0.88, 2.41)
9	1.27 (0.81, 2.00)	1.61 (0.97, 2.62)
10 (Most deprived)	1.66 (1.08, 2.54)	2.10 (1.32, 3.40)
11 (Missing)	1.24 (0.60, 2.60)	1.77 (0.80, 3.55)
*Body mass index (kg/m*^*2*^*)*		
Normal weight (18.5–24.9)	Reference	
Underweight (< 18.5)	1.12 (0.54, 2.15)	0.98 (0.47, 2.05)
Overweight (25–29.9)	1.28 (0.98, 1.68)	0.92 (0.68, 1.32)
Obesity class I (30–34.9)	0.87 (0.56, 1.36)	1.34 (0.93, 1.94)
Obesity class II (35–39.9)	1.24 (0.68, 2.28)	0.98 (0.52, 1.83)
Obesity class III (> 40)	1.13 (0.52, 2.45)	0.72 (0.30, 1.78)
Missing	0.83 (0.63, 1.10)	0.83 (0.63, 1.10)
*Smoking status*		
Never/Non-smoker	Reference	
Current Smoker	1.20 (0.94, 1.52)	1.18 (0.92, 1.52)
Ex-smoker	0.92 (0.56, 1.50)	0.78 (0.45, 1.34)
Missing	0.93 (0.70, 1.26)	0.99 (0.73, 1.33)

##### Study participants aged 0–16 years at index date

At index date, 12.30% and 12.60% of HSP and matched-control study participants were aged 0–16 years old. At baseline, there was no depression in both the HSP and matched-control group. Less than 5 people in the HSP and matched-control group had anxiety, respectively.

At follow up, the incidence for depression was 5.59% and 4.30% in the HSP and matched-control group, respectively. For those with depression at follow up, the mean age at index date was 10 and 11 years old for the HSP and matched-control group, respectively; mean years of follow up from index date was 10 and 11 years for the HSP and matched-control group, respectively.

At follow up, the incidence for anxiety was 9.09% and 5.30% in the HSP and matched-control group, respectively. For those with anxiety at follow up, the mean age at index date were both 10 years old for the HSP and matched-control group; mean years of follow up from index date was 9 and 10 years for the HSP and matched-control group, respectively (Appendix 7).

## Discussion

### Main findings

This observational study used primary care routine health records to describe the epidemiology of HSP in England and Northern Ireland from 2000 to 2021. We also studied the association between HSP and common mental health conditions (depression and anxiety). HSP prevalence increased from 2.83 per 100,000 population in 2000 to 6.27 per 100,000 population in 2021; whilst HSP incidence remained stable and was 0.29 per 100,000 person-years in 2021. Having a diagnosis of HSP was associated with higher risk of a diagnosis of depression or anxiety.

### Findings in the context of existing literature

The prevalence of HSP in this study (6.27 per 100,000 population in 2021) is higher than the global prevalence modelled by Stichele et al. 2022 (3.6 per 100,000) using existing literature, systematic reviews and expert opinion [5]. Compared to two other cross-sectional studies in European countries with similar health care system, our prevalence was higher than the 2.24 per 100,000 reported by Suero et all in Spain (2018–2019) but similar to the 7.4 per 100,000 reported by Erichsen et al. in Southeast Norway (2002–2008) [6, 9].

Both these studies recruited HSP patients through relevant clinicians and patient associations. The Norwegian study additionally used hospital records and genetic databases and recruited potentially affected family members through index cases; this comprehensive recruitment strategy is likely to have contributed to the higher prevalence reported.

The association between HSP and mental ill health in this study is consistent with previous research [18, 26–28] Marvel et al. (2024), which demonstrated substantial rates of anxiety (23%) and depression (12.6%) in childhood-onset HSP patients found higher rates of anxiety (23%) and depression (12.6%) in childhood-onset HSP patients compared to the general paediatric population. The study also reported that children were anxious in situations where they knew they had to do a lot of walking, especially in public places [26]. Related to mental well-being, studies have also shown that HSP patients have lower health related quality of life compared to the general population, with higher disease severity correlating with worse quality of life [17, 29]. Kersten et al.’s qualitative study on the experience of patients with pure form HSP captured the struggles with HSP motor symptoms, limiting physical activities and social participation, resulting in negative emotions including fear, frustration and depression [16].

### Strengths and limitations

Previous European population based cross sectional studies recruited HSP patients through hospital specialist such as neurologists or paediatricians [6, 9]. This relies on the clinicians having a list of HSP patients. HSP patients who are not being regularly followed up in specialist clinics may be missed [9]. As most of the UK population are registered with a general practitioner, who acts as the gate keeper to specialist services, primary care records is a good longitudinal data source to study long-term conditions [11]. This enabled our study to not only provide a point prevalence, but also the trend of HSP prevalence and incidence over two decades.

However, this study was unable to ascertain the HSP genotype using primary care routine health records. Linkage to genetic laboratory test results is not available. This study is not able to ascertain whether the HSP diagnosis was genetically confirmed. However, referral to the genetics team for a genetic test would usually be part of the diagnostic assessment. As more than half of clinically suspected HSP cases do not have a pathogenic variant [1], it is likely the HSP cohort in this study represents those with and without a genetic confirmation following a genetic test. This meant that although we observed higher odds of a recorded HSP diagnosis in patients who are male, of white ethnicity, and reside outside of London, we are unable to make further inference. Omidvar et al.’s 2019 systematic review found that gender distribution in HSP differed by genotype, with predominance of male patients in certain genotype, e.g., SPG7 [30]. The genetic penetrance also relies on a patient’s sex for certain genotypes (e.g., SPG4) [31].

Current SNOMED-CT diagnostic codes used in general practice health records do not distinguish between the pure and complex subtypes of HSP. Although this study has shown higher incidence of anxiety and depression in all types of HSP compared to people without HSP, we were not able to show whether the common mental health outcomes differ by HSP subtypes. Studies have shown that lower mobility levels correlated with worse health related quality of life (including the mental health component) and Beck Depression Inventory score in HSP patients. [18, 29].

The diagnostic odyssey for rare disease means there is often a delay from symptom onset to a patient receiving a diagnosis [32, 33]. Saputra et al.’s paper discussed the challenges with getting a clinical and genetic diagnosis for HSP, including the phenotypic overlap of HSP with other disorders [34]. A study from 18 HSP patients found a mean delay of 5.8 years from symptom onset to a genetic diagnosis [35]. Mild symptoms at initial onset may not be recognised to be related to HSP [9]. This meant that in primary care records, the first event date related to a HSP diagnosis code may not be a good proxy for age of onset. Therefore, for this study, we only have age at index date and could not characterise the HSP cohort by age of onset. It also meant that the temporality of the common mental health outcome in relation to HSP onset is less clear in this study. Nevertheless, the mental stress related to the diagnostic odyssey would predate the diagnosis date (which may be more closely related to study index date). Regardless, the rates of depression and anxiety were higher both at baseline and after study index date.

Limitations that are inherent with using routine health records as the data source applies to this study, including missing data for ethnicity, BMI and smoking data. Routine health records capture a diagnosis only when a patient is in contact with the health services. Inequality in accessing health services in certain sociodemographic group may result in underrepresentation in the HSP cohort, such as people from deprived socioeconomic or ethnic minority background. However, this underrepresentation is less likely to affect severe health conditions with high health and social care needs, particularly in the NHS where health care is free. [36, 37].

There may be residual confounding from variables that were not available and not adjusted for. Although generally considered representative of the UK population, the CPRD dataset used in this analysis covers 24% of the UK population.

### Clinical and research implications

The literature shows that HSP patients generally have normal life expectancy [5]. Therefore the rise in prevalence and stable incidence of HSP over the last 2 decades observed in this study is likely to reflect the improvement of life expectancy in the general population. This information on rising HSP prevalence is important for planning health care services to ensure current and future provision meet the needs of a growing HSP population.

The higher rates of anxiety and depression amongst HSP patients compared to non HSP patients illustrates the importance of incorporating mental health in the management of HSP patients. When presented with this study finding, members of the UK HSP support group reported their mental health concerns have not been explored by healthcare providers. The England Rare Diseases Action Plan recognises the challenges with access to mental health and psychological support for people living with rare diseases and has subsequent action plans to address this [38].

Most HSP epidemiological studies used surveys of clinicians or patient associations and hospital records as the data source [39]. Surveys can be subjected to responder bias. HSP patients, once diagnosed by neurologist or geneticist, are often discharged from follow up, with supportive management provided by allied health professionals or neurorehabilitation teams, limiting the utility of inpatient hospital records for HSP epidemiological studies [33]. This study demonstrated the utility of using routine primary care health records for epidemiological studies of rare diseases that are predominantly managed in the community. It will serve as a useful comparator for descriptive epidemiology from national registries that uses hospital and genetic laboratory as data sources.

This study focused on all types of HSP using SNOMED-CT diagnostics codes in general practice health records. The prevalence reported in this study is higher than globally modelled prevalence. Nevertheless, it is important to consider the possibility of missed cases of HSP through future epidemiological studies that includes phenotypic mimics. Future studies could also explore the utility of phenotypic algorithm using symptoms-based codes to differentiate between the HSP subtypes. To help quantify the disease burden and help with service planning for HSP patients in the UK, further studies could utilise primary care data linked to hospital and mortality data to examine mortality, co-morbidities and hospital admissions in HSP patients.

## Conclusion

This study provided the first descriptive epidemiology for HSP in England and Northern Ireland, demonstrating the utility of primary care routine health records for studying rare diseases. The higher rates of common mental health conditions in HSP patients illustrated the importance of access to mental health support. Further studies using primary care data linked to other routine dataset will improve the understanding of the health profile of HSP patients in the UK.

## Data Availability

The datasets analysed during the current study are not publicly available due to CPRD licensing restrictions. However, access can be obtained from the Clinical Practice Research Datalink (www.cprd.com) subject to standard application procedures.

## Appendix

### Appendix 1. SNOMED CT codes for HSP

**Table:**

MEDICAL_CODE_ID	DESCRIPTION	SNOMED_CT_CODE
66483014	Hereditary spastic paraplegia	39912006
3142321000006110	Familial spastic paraplegia syndrome	39912006
3142311000006110	Strumpell-Lorrain disease	39912006
3142331000006110	HSP—Hereditary spastic paraplegia	39912006
3142351000006110	Spastic congenital paraplegia	39912006
3142341000006110	Strumpell disease	39912006
7848031000006110	Autosomal recessive spastic paraplegia type 55	723825006
13621061000006100	Autosomal dominant spastic paraplegia type 3	782670003

### Appendix 2. SNOMED CT codes for depression

**Table:**

MEDICAL_CODE_ID	DESCRIPTION	SNOMED_CT_CODE
138421012	Agitated depression	83458005
294646011	Senile dementia with depression	191459006
294655014	Arteriosclerotic dementia with depression	191466007
294828015	Single major depressive episode, severe, with psychosis	191604000
294837015	Recurrent major depressive episodes, mild	191610000
294838013	Recurrent major depressive episodes, moderate	191611001
294840015	Recurrent major depressive episodes, severe, with psychosis	191613003
294917018	Reactive depressive psychosis	191676002
294918011	Psychotic reactive depression	191676002
295536013	Postviral depression	192079006
346972018	Endogenous depression first episode	231499006
346973011	Masked depression	231500002
369982012	Seasonal affective disorder	247803002
410861011	Endogenous depression—recurrent	274948002
1773563015	Depression management programme	401174001
2474715017	On depression register	413169006
2533375017	Patient given advice about management of depression	415044007
2534091015	Depression interim review	413973005
2534092010	Depression medication review	413974004
2534096013	Depression annual review	413972000
213641000000111	[X]Mild depression	310495003
300711000000119	Referral for guided self-help for depression	199111000000100
408521000000112	Depression monitoring first letter	717211000000107
408561000000116	Depression monitoring third letter	716961000000102
681801000000113	On full dose long term treatment for depression	361761000000106
853871000006111	Post natal depression	853871000006107
882401000006115	Reactive (neurotic) depression	87414006
908731000006114	[RFC] Postnatal depression	908731000006105
1771531000006110	Antenatal depression	1771531000006100
1972071000006110	Unspecified dementia, other symptoms, predominantly depressive	1972071000006100
1972111000006110	Organic depressive disorder	1972111000006100
1972311000006110	Dementia in Alzheimer's dis, atypical or mixed type, other symptoms, predominantly depressive	1972311000006100
1972451000006110	Dementia in Alzheimer's disease, unspecified, other symptoms, predominantly depressive	1972451000006100
1972541000006110	Vascular dementia of acute onset, other symptoms, predominantly depressive	1972541000006100
1972911000006110	Mixed cortical and subcortical vascular dementia, other symptoms, predominantly depressive	1972911000006100
1973381000006110	Other vascular dementia, other symptoms, predominantly depressive	1973381000006100
1975191000006110	Post-schizophrenic depression, continuous	1975191000006100
1975211000006110	Post-schizophrenic depression, episodic with progressive deficit	1975211000006100
1975261000006110	Post-schizophrenic depression, episodic remittent	1975261000006100
1975321000006110	Post-schizophrenic depression, course uncertain, period of observation too short	1975321000006100
1975981000006110	Mild depressive episode, without somatic syndrome	1975981000006100
1976021000006110	Moderate depressive episode, without somatic syndrome	1976021000006100
1976231000006110	Recurrent depressive disorder, current episode mild, with somatic syndrome	1976231000006100
1976271000006110	Recurrent depressive disorder, current episode moderate, with somatic syndrome	1976271000006100
1976491000006110	Mixed anxiety and depressive reaction	1976491000006100
1976921000006110	Post-schizophrenic depression, other	1976921000006100
2620391000000110	Maternal postnatal depression	1038261000000100
2659601000000110	Signposting to depression self-help group	1057351000000100
294647019	Senile dementia with depressive or paranoid features NOS	191457008
294836012	Recurrent major depressive episodes, unspecified	268621008
295494011	Brief depressive reaction NOS	192046006
295535012	Depressive disorder NEC	35489007
296199015	[X]Other recurrent depressive disorders	191616006
401871010	[X]Other depressive episodes	35489007
401872015	[X]Depressive episode, unspecified	35489007
401873013	[X]Recurrent depressive disorder	191616006
401876017	[X]Recurrent depressive disorder, unspecified	191616006
202561000006114	Prolonged depressive reaction	192049004
359121000006116	[X] Reactive depression NOS	87414006
376701000006116	[X]Depressive conduct disorder	231542000
376721000006114	[X]Depressive episode	35489007
376741000006119	[X]Depressive neurosis	78667006
379431000006113	[X]Dysthymia	78667006
398351000006110	[X]Mild anxiety depression	231504006
398561000006117	[X]Mixed anxiety and depressive disorder	231504006
398841000006119	[X]Monopolar depression NOS	35489007
399961000006118	[X]Neurotic depression	78667006
408601000000116	Depression monitoring telephone invite	716421000000103
423041000006119	[X]Post-schizophrenic depression	231485007
423131000006113	[X]Postnatal depression NOS	58703003
423141000006115	[X]Postpartum depression NOS	58703003
424551000006113	[X]Recurr severe episodes/psychogenic depressive psychosis	191613003
424561000006110	[X]Recurrent brief depressive episodes	40568001
424571000006115	[X]Recurrent depress disorder cur epi severe with psyc symp	28475009
424631000006119	[X]Recurrent episodes of depressive reaction	191616006
424641000006112	[X]Recurrent episodes of psychogenic depression	191616006
424651000006114	[X]Recurrent episodes of reactive depression	191616006
425501000006113	[X]Schizoaffective disorder, depressive type	84760002
425561000006114	[X]Schizoaffective psychosis, depressive type	84760002
425751000006115	[X]Seasonal depressive disorder	247803002
426931000006117	[X]Single episode of masked depression NOS	231500002
426971000006119	[X]Single episode of reactive depression	87414006
488211000006112	Anxiety with depression	231504006
525921000006119	Brief depressive reaction	192046006
613791000006115	Depressive psychoses	35489007
1715191000006110	[X]Major depression, severe with psychotic symptoms	73867007
1715781000006110	[X]Major depression, severe without psychotic symptoms	75084000
1785881000006110	[X]Antenatal depression	790961000000101
294845013	Recurrent major depressive episode NOS	268621008
296180012	[X]Recurrent depressive disorder, current episode mild	310495003
296181011	[X]Recurrent depressive disorder, current episode moderate	310496002
1222477019	[D]Postoperative depression	82218004
182771000006112	Recurrent major depressive episodes, severe, no psychosis	764611000000100
419841000006116	[X]Persistant anxiety depression	231504006
423611000006111	[X]Prolonged single episode of reactive depression	87414006
426941000006110	[X]Single episode of psychogenic depression	87414006
426981000006116	[X]Single episode of reactive depressive psychosis	191676002
296137015	[X]Mild depressive episode	310495003
296138013	[X]Moderate depressive episode	310496002
401766011	Single major depressive episode	36923009
401866015	[X]Severe depressive episode without psychotic symptoms	310497006
223741000000112	[X]Single episode major depression w'out psychotic symptoms	310497006
424681000006118	[X]Recurrent severe episodes/reactive depressive psychosis	1086471000000100
142541000006115	Single major depressive episode, severe, without psychosis	251000119105
6000691000006110	Mild depression	310495003
3094951000006110	Major depressive disorder, single episode	36923009
4539971000006110	History of depression	161469008
12451461000006100	Single major depressive episode, moderate	15639000
6550791000006110	Counseling for postnatal depression	395072006
11921141000006100	[X]Recurrent severe episodes/reactive depressive psychosis	191613003
3700911000006110	Psychotic depression	73867007
6000711000006110	Moderate depression	310496002
6550801000006110	Counselling for postnatal depression	395072006
5024511000006110	Depressive conduct disorder	231542000
3837121000006110	Postoperative depression	82218004
6649631000006110	Depression management program	401174001
8464961000006110	Recurrent reactive depressive episodes, severe, with psychosis	1086471000000100
2957311000006110	Severe recurrent major depression with psychotic features	28475009
3878611000006110	Schizophreniform psychosis, depressive type	84760002
3071791000006110	Depressive illness	35489007
3153071000006110	Recurrent brief depressive disorder	40568001
5023901000006110	Post-schizophrenic depression	231485007
4539951000006110	History of depressive disorder	161469008
12727931000006100	Depressive disorder NEC	609311000000100
882671000006112	Depression	609311000000100
882681000006110	Depression NOS	609311000000100
882821000006110	Moderate depression	465441000000108
882831000006113	Severe depression	397701000000102
8245201000006110	Depression care management	784051000000106
11918531000006100	[X]Mild depressive episode	430421000000104
2391541000000110	Referral for guided self-help for depression declined	933441000000101
1972131000006110	Dementia in Alzheimer's disease with early onset, other symptoms, predominantly depressive	1972131000006100
1972661000006110	Multi-infarct dementia other symptoms, predominantly depressive	1972661000006100
1976251000006110	Recurrent depressive disorder, current episode moderate, without somatic syndrome	1976251000006100
294621000000118	Depression resolved	196381000000100
294894013	Atypical depressive disorder	191659001
226581000000119	Depression—enhanced service completed	166481000000107
376691000006116	[X]Depression NOS	35489007
376711000006118	[X]Depressive disorder NOS	35489007
424541000006111	[X]Recurr severe episodes/major depression + psychotic symptom	28475009
425411000006110	[X]SAD—Seasonal affective disorder	247803002
675861000006113	Neurotic depression reactive type	87414006
1680571000006110	On full dose long term treatment depression—enh serv admin	361761000000106
401869010	[X]Severe depressive episode with psychotic symptoms	191604000
379771000006116	[X]Endogenous depression with psychotic symptoms	73867007
396081000006116	[X]Major depression, recurrent without psychotic symptoms	268621008
424531000006118	[X]Recurr depress disorder cur epi severe without psyc sympt	310497006
426921000006115	[X]Single episode of major depression and psychotic symptoms	191604000
426951000006112	[X]Single episode of psychogenic depressive psychosis	191676002
6000721000006110	Severe depression	310497006
12491591000006100	Depression care management	784051000000106
11921301000006100	[X]Severe depressive episode without psychotic symptoms	397701000000102
12626331000006100	QOF (Quality and Outcomes Framework) depression quality indicator-related care invitation	1110911000000100
2370061000000110	Referral for depression self-help video	923921000000104
1972201000006110	Dementia in Alzheimer's disease with late onset, other symptoms, predominantly depressive	1972201000006100
1972771000006110	Subcortical vascular dementia, other symptoms, predominantly depressive	1972771000006100
1975991000006110	Mild depressive episode with somatic syndrome	1975991000006100
1976051000006110	Moderate depressive episode, with somatic syndrome	1976051000006100
1976211000006110	Recurrent depressive disorder, current episode mild, without somatic syndrome	1976211000006100
1823881000006110	Depression confirmed	1823881000006100
909681000006110	[RFC] Depression	909681000006106
939961000006116	Postnatal depression discussed	939961000006100
406881000000111	Depression monitoring administration	713831000000108
408541000000117	Depression monitoring second letter	716681000000100
441826016	Endogenous depression	300706003
294690018	Drug-induced depressive state	191495003
294844012	Recurrent depression	191616006
223651000000118	Postnatal depression	58703003
408581000000113	Depression monitoring verbal invite	717261000000109
424671000006116	[X]Recurrent severe episodes of psychotic depression	191613003
474171000006112	Agitated depression	83458005
642461000006116	Endogenous depression first episode	231499006
1715181000006110	[X]Major depression, moderately severe	832007
1715771000006110	[X]Major depression, mild	87512008
379781000006118	[X]Endogenous depression without psychotic symptoms	300706003
432511000006119	[X]Vital depression recurrent without psychotic symptoms	310497006
294825017	Single major depressive episode, mild	79298009
294826016	Single major depressive episode, moderate	15639000
294824018	Single major depressive episode, unspecified	36923009
426991000006118	[X]Single episode vital depression w'out psychotic symptoms	310497006
3087971000006110	Severe recurrent major depression without psychotic features	36474008
4763361000006110	Drug-induced depression	191495003
7965131000006110	Severe major depression, single episode	251000119105
1973551000006110	Vascular dementia, unspecified other symptoms, predominantly depressive	1973551000006100
1975231000006110	Post-schizophrenic depression, episodic with stable deficit	1975231000006100
1976411000006110	Other recurrent mood affective disorders, recurrent brief depressive disorder	1976411000006100
2748031000006110	Moderate major depression, single episode	15639000
1231868010	Puerperal depression	58703003
1488771018	Postnatal depression counselling	395072006
294642013	Presenile dementia with depression	191455000
294644014	Senile dementia with depressive or paranoid features	191457008
295537016	Chronic depression	192080009
251629019	H/O: depression	161469008
182721000006111	Recurrent major depressive episode	268621008
226411000000110	Depression—enhanced services administration	166291000000108
3094941000006110	Major depression, single episode	36923009
425691000006119	[X]Schizophreniform psychosis, depressive type	84760002
426961000006114	[X]Single episode of psychotic depression	191604000
366561000006119	[X]Atypical depression	191659001
426911000006111	[X]Single episode of depressive reaction	87414006
294832014	Single major depressive episode NOS	36923009
426891000006114	[X]Single episode agitated depressn w'out psychotic symptoms	310497006
6550781000006110	Postnatal depression counseling	395072006
5024071000006110	Anxiety depression	231504006
3789581000006110	Mild major depression, single episode	79298009
11918561000006100	[X]Moderate depressive episode	465441000000108
882811000006119	Mild depression	430421000000104

### Appendix 3. SNOMED CT codes for anxiety

**Table:**

MEDICAL_CODE_ID	DESCRIPTION	SNOMED_CT_CODE
65032014	Dental phobia	38617005
90724016	Simple phobia	54587008
97986013	Acrophobia	58963008
251630012	H/O: anxiety state	161470009
294961014	Recurrent anxiety	191709001
294994012	Social phobia, fear of eating in public	191724005
294995013	Social phobia, fear of public speaking	191725006
342665019	Anxiety management training	228560001
370049015	Flying phobia	247854002
481154010	Generalised anxiety disorder	21897009
485870016	Cancer phobia	34563004
1210253015	Panic disorder	371631005
1231321013	Animal phobia	54307006
2533632018	H/O: agoraphobia	414371008
300681000000118	Referral for guided self-help for anxiety	199101000000102
516601000000113	Anxious	48694002
851141000006112	Phonophobia	851141000006108
851351000006112	School phobia	851351000006108
909691000006113	[RFC] Anxiety management	909691000006109
932131000006118	Needle phobia	54587008
972931000006117	** The treatment of anxiety disorders	972931000006101
1808521000006110	Panic disorder without agoraphobia	56576003
2391581000000110	Referral for guided self-help for anxiety declined	933461000000100
2618361000000110	Referral for psychological management of anxiety	1037451000000100
294953016	Anxiety state unspecified	198288003
294963012	Anxiety state NOS	198288003
294993018	Agoraphobia without mention of panic attacks	61569007
296224013	[X]Phobic anxiety disorders	386810004
296237011	[X]Phobic anxiety disorder, unspecified	386810004
296238018	[X]Other anxiety disorders	197480006
296722013	[X]Separation anxiety disorder of childhood	11806006
362871000006111	[X]Agoraphobia	70691001
362881000006114	[X]Agoraphobia without history of panic disorder	70691001
363281000006117	[X]Animal phobias	54307006
363641000006114	[X]Anxiety hysteria	197480006
363651000006111	[X]Anxiety neurosis	207363009
370861000006110	[X]Childhood overanxious disorder	13438001
378291000006117	[X]Dream anxiety disorder	419145002
388071000006116	[X]Generalized anxiety disorder	21897009
398351000006110	[X]Mild anxiety depression	231504006
398561000006117	[X]Mixed anxiety and depressive disorder	231504006
400201000006111	[X]Nosophobia	18193002
403931000006116	[X]Organic anxiety disorder	17496003
418061000006116	[X]Panic state	371631005
427051000006113	[X]Social anxiety disorder of childhood	64165008
427071000006115	[X]Social phobias	25501002
488211000006112	Anxiety with depression	231504006
626101000006114	Disturbance anxiety and fearfulness childhood/adolescent NOS	192108001
980191000006111	[X]Needle phobia	231501003
296236019	[X]Other phobic anxiety disorders	386810004
419841000006116	[X]Persistant anxiety depression	231504006
426871000006113	[X]Simple phobia	386810004
488201000006114	Anxiety states	197480006
7663271000006110	Management of anxiety	710060004
3265831000006110	Adjustment disorder with anxiety	47372000
4981541000006110	AMT—Anxiety management training	228560001
6857231000006110	History of agoraphobia	414371008
3649691000006110	Phobia of going out	70691001
4539991000006110	History of anxiety state	161470009
3265811000006110	Adjustment disorder with anxious mood	47372000
6358151000006110	Episodic paroxysmal anxiety disorder	371631005
11920891000006100	[X]Phobia NOS	386810004
2907991000006110	Social anxiety disorder	25501002
3287761000006110	Feeling anxious	48694002
3785111000006110	Anxious cognitions	79015004
6025531000006110	Anxiety counseling	313087008
2848311000006110	GAD—Generalised anxiety disorder	21897009
3287741000006110	Anxiety	48694002
3385931000006110	Isolated phobia	54587008
6025541000006110	Counseling for anxiety	313087008
2848291000006110	Generalized anxiety disorder	21897009
2848301000006110	GAD—Generalized anxiety disorder	21897009
988881000006118	Agoraphobia	191723004
991621000006112	Needle phobia	563201000000101
12716981000006100	Agoraphobia without mention of panic attacks	191723004
2618401000000110	Referral for psychological management of anxiety declined	1037471000000100
1818111000006110	Feeling anxious	1818111000006100
882381000006115	Agoraphobia	191722009
253620013	O/E—anxious	162723006
296249012	[X]Anxiety disorder, unspecified	197480006
362181000006117	[X]Acrophobia	54587008
363661000006113	[X]Anxiety NOS	197480006
420641000006116	[X]Phobic anxiety disorder of childhood	192611004
626121000006116	Disturbance of anxiety and fearfulness childhood/adolescent	192108001
6025551000006110	Counselling for anxiety	313087008
4550971000006110	On examination—anxious	162723006
294991016	Phobia unspecified	386808001
296239014	[X]Panic disorder [episodic paroxysmal anxiety]	371631005
1861181000006110	Breathlessness causing anxiety	1861181000006100
853241000006119	Phobic anxiety	853241000006103
294960010	Chronic anxiety	191708009
294996014	Social phobia, fear of public washing	191726007
2777721000006110	Organic anxiety disorder	17496003
363361000006117	[X]Anthropophobia	25501002
363691000006117	[X]Anxious [avoidant] personality disorder	231528008
418051000006118	[X]Panic disorder with agoraphobia	191722009
2712981000006110	Overanxious disorder of childhood	13438001
3381271000006110	Zoophobia	54307006
296245018	[X]Other mixed anxiety disorders	231504006
2618241000000110	Anxiety resolved	1037391000000100
441512015	Separation anxiety disorder	11806006
294992011	Agoraphobia with panic attacks	191722009
33475016	Claustrophobia	19887002
401881014	[X]Other specified anxiety disorders	197480006
223641000000116	Phobic anxiety	386810004
4767501000006110	Childhood phobic anxiety disorder	192611004
363681000006115	[X]Anxiety state	198288003
371071000006117	[X]Claustrophobia	54587008
420631000006114	[X]Phobia NOS	386810004
427231000006111	[X]Specific (isolated) phobias	54587008
6025521000006110	Anxiety counselling	313087008
5024071000006110	Anxiety depression	231504006
4764811000006110	Cyesiophobia	191733007
12703831000006100	[X]Organic anxiety disorder	17496003
882391000006117	Other phobias	563201000000101
304838011	Anxiety state	198288003

### Appendix 4. HSP prevalence per 100,000 person-years

**Table:**

Year	Prevalence Per 100,000 Population (95% Confidence Interval)	Numerator (Total Cases)	Denominator (Total Population)
2000	2.83 (2.49, 3.20)	254	8,985,873
2001	2.87 (2.54, 3.24)	265	9,233,071
2002	3.11 (2.77, 3.49)	295	9,479,875
2003	3.33 (2.97, 3.71)	322	9,672,880
2004	3.46 (3.10, 3.84)	342	9,891,136
2005	3.64 (3.28, 4.04)	364	9,990,272
2006	3.88 (3.51, 4.28)	395	10,179,087
2007	3.90 (3.53, 4.30)	403	10,334,465
2008	4.31 (3.92, 4.72)	453	10,511,255
2009	4.37 (3.99, 4.79)	467	10,676,610
2010	4.50 (4.11, 4.92)	488	10,851,073
2011	4.47 (4.08, 4.88)	490	10,966,738
2012	4.50 (4.12, 4.91)	500	11,109,256
2013	4.75 (4.35, 5.17)	535	11,264,909
2014	4.96 (4.55, 5.39)	552	11,138,746
2015	5.26 (4.84, 5.70)	593	11,284,513
2016	5.44 (5.03, 5.89)	629	11,555,718
2017	5.47 (5.05, 5.90)	646	11,821,550
2018	5.68 (5.26, 6.12)	688	12,114,586
2019	5.82 (5.40, 6.26)	715	12,295,424
2020	6.08 (5.65, 6.54)	744	12,230,999
2021	6.27 (5.83, 6.73)	759	12,113,119

### Appendix 5. Incidence rate of HSP

**Table:**

Year	Incidence Rate Per 100,000 Person Years (95% Confidence Interval)	Numerator (new cases)	Denominator (population at risk)
2000	0.12 (0.06, 0.22)	11	9,091,024
2001	0.15 (0.08, 0.25)	14	9,354,477
2002	0.29 (0.19, 0.42)	28	9,566,519
2003	0.25 (0.16, 0.37)	24	9,772,024
2004	0.26 (0.17, 0.38)	26	9,931,256
2005	0.28 (0.19, 0.40)	28	10,076,997
2006	0.19 (0.11, 0.29)	19	10,240,497
2007	0.30 (0.20, 0.42)	31	10,405,416
2008	0.20 (0.12, 0.30)	21	10,591,616
2009	0.19 (0.12, 0.30)	21	10,756,776
2010	0.18 (0.11, 0.28)	20	10,892,085
2011	0.23 (0.15, 0.34)	25	11,015,021
2012	0.30 (0.20, 0.42)	33	11,174,010
2013	0.16 (0.10, 0.26)	18	11,162,383
2014	0.27 (0.18, 0.38)	30	11,187,922
2015	0.25 (0.17, 0.37)	29	11,402,001
2016	0.31 (0.22, 0.43)	36	11,674,034
2017	0.39 (0.28, 0.51)	46	11,960,506
2018	0.35 (0.26, 0.47)	43	12,213,115
2019	0.31 (0.22, 0.43)	38	12,223,814
2020	0.31 (0.22, 0.43)	38	12,140,036
2021	0.29 (0.20, 0.40)	34	11,941,789

### Appendix 6. Logistic regression for baseline depression and anxiety for people with HSP compared to people without HSP

**Table:**

	DEPRESSION	ANXIETY
Variables	OR (95% CI)	OR (95% CI)
Exposed (HSP)	1.74 (1.48, 2.06)	1.31 (1.08, 1.60)
*Age Category (in years)*		
0–16	Reference	
17–30	0.65 (0.38, 1.14)	6.87 (3.08, 15.30)
31–40	1.14 (0.67, 1.95)	6.55 (2.92, 14.68)
41–50	1.45 (0.85, 2.48)	9.91 (4.47, 21.97)
51–60	1.53 (0.87, 2.70)	11.32 (5.09, 25.17)
61–70	1.12 (0.65, 1.92)	8.83 (3.94, 19.76)
71–80	1.27 (0.72, 2.24)	10.31 (4.50, 23.62)
81+	1 (omitted)	6.79 (2.44, 18.91)
*Sex*		
Male	Reference	
Female	2.04 (1.76, 2.36)	1.74 (1.47, 2.05)
*Ethnicity*		
Asian	0.70 (0.47, 1.03)	0.58 (0.37, 0.95)
Black	0.28 (0.12, 0.65)	0.12 (0.03, 0.52)
Mixed	0.50 (0.20, 1.27)	0.90 (0.38, 2.16)
Other	0.55 (0.16, 1.88)	0.71 (0.21, 2.36)
White	Reference	
Missing	0.79 (0.67, 0.94)	0.65 (0.53, 0.80)
*Index of Multiple Deprivation*		
1 (Least deprived)	Reference	
2	1.17 (0.82, 1.65)	0.90 (0.61, 1.32)
3	1.51 (1.07, 2.11)	0.95 (0.65, 1.40)
4	1.43 (1.02, 1.99)	1.08 (0.75, 1.55)
5	1.44 (1.02, 2.03)	1.00 (0.68, 1.47)
6	1.46 (1.02, 2.04)	1.09 (0.76, 1.57)
7	1.35 (0.96, 1.90)	1.09 (0.76, 1.58)
8	1.51 (1.08, 2.12)	1.32 (0.92, 1.89)
9	1.42 (1.01, 2.00)	1.26 (0.87, 1.81)
10 (Most deprived)	1.79 (1.29, 2.50)	1.12 (0.78, 1.61)
11 (Missing)	1.69 (0.98, 2.90)	1.01 (0.50, 2.03)
*Body Mass Index (kg/m2)*		
Normal weight (18.5–24.9)	Reference	
Underweight (< 18.5)	1.40 (0.87, 2.26)	1.79 (1.09, 2.94)
Overweight (25–29.9)	0.96 (0.79, 1.18)	0.88 (0.70, 1.09)
Obesity class I (30–34.9)	1.42 (1.13, 1.80)	1.12 (0.85, 1.46)
Obesity class II (35–39.9)	1.65 (1.18, 2.32)	1.09 (0.73, 1.64)
Obesity class III (> 40)	1.49 (0.98, 2.25)	1.14 (0.70, 1.85)
Missing	0.63 (0.50, 0.80)	0.71 (0.55, 0.92)
*Smoking Status*		
Current Smoker	1.66 (1.40, 1.96)	1.55 (1.29, 1.88)
Never/Non-smoker	Reference	
Ex-smoker	1.12 (0.79, 1.58)	0.71 (0.45, 1.12)
Missing	0.85 (0.64, 1.12)	0.73 (0.53, 1.01)

### Appendix 7. Common mental health outcomes for participants aged 0–16 at index date

**Table:**

Common mental health outcomes	HSP	Matched-control
Baseline prevalence		
Depression	0	0
Anxiety	< 5	< 5
Outcome incidence		
Depression	5.59% (10/179)	4.30% (31/721)
Anxiety	9.09% (16/176)	5.30% (38/717)
Mean age at index date for people with the mental health outcomes, in years		
Depression	10.31	11.02
Anxiety	10.45	10.01
Mean years of follow up from index date for people with the mental health outcomes		
Depression	9.67	10.54
Anxiety	9.37	10.03

### Appendix 8. STROBE-RECORD checklist

**Table:**

Item No	STROBE/RECORD Items	Page No
1	Indicate study design with a common term in the title or abstract	2
1.1	Specify type of data used in title or abstract; include database names if possible	2
1.2	Report geographic region and timeframe in title or abstract	2
1.3	If linkage between databases occurred, state it in title or abstract	2
2	Explain scientific background and rationale	4
3	State specific objectives and hypotheses	4
4	Present key elements of study design early in the paper	5
5	Describe setting, locations, and dates of data collection	5
6	Give eligibility criteria and methods for participant selection; include follow-up	6
6.1	List methods of study population selection (codes/algorithms), if possible	6
6.2	Reference validation studies of codes/algorithms, or provide details if conducted in this study	7
6.3	Include a flow diagram to illustrate data linkage process	9
7	Clearly define outcomes, exposures, predictors, confounders, and effect modifiers	7,8
7.1	List codes/algorithms for exposures, outcomes, confounders, and effect modifiers; provide explanation if unavailable	26–50
8	Give data sources and assessment methods for each variable	7
9	Describe efforts to address potential sources of bias	21,22
10	Explain rationale for study size	5
11	Explain handling of quantitative variables in analysis, and groupings if applied	7
12	Describe statistical methods, including for confounding control	8
12.1	Describe extent of investigator access to database population used to create study population	7
12.2	Describe data cleaning methods	6
12.3	State whether data linkage occurred and provide linkage quality evaluation methods	6
13	Report participant numbers at each study stage, reasons for non-participation, and consider flow diagram use	8,9
13.1	Detail study population selection, including filtering criteria based on data quality, data availability, and linkage	8,9,10
14	Provide participant characteristics and data on exposures and confounders; indicate missing data	10,17
15	Report numbers of outcome events or summary measures	21
16	Provide unadjusted and confounder-adjusted estimates, category boundaries, and absolute risk if relevant	11–15
17	Report subgroup, interaction, and sensitivity analyses	-
18	Summarize key results with reference to objectives	21
19	Discuss limitations, including potential bias or imprecision	22
19.1	Discuss implications of using data not collected to answer the specific research question	23,24
20	Provide a cautious interpretation of results considering objectives and evidence	17,18
21	Discuss generalizability of study results	21
22	State funding sources and funder roles	25
22.1	Provide accessibility information for protocol, raw data, or programming code	25

## Abbreviations

BMI: Body mass index
CI: Confidence interval
CPRD: Clinical practice research datalink
HR: Hazards ratio
HSP: Hereditary spastic paraplegia
OR: Odds ratio
NHS: National Health Service
SNOMED-CT: Systematized nomenclature of medicine-clinical terms
